# DNA methylation landscapes from pig’s limbic structures underline regulatory mechanisms relevant for brain plasticity

**DOI:** 10.1038/s41598-022-20682-x

**Published:** 2022-09-29

**Authors:** Alvaro Perdomo-Sabogal, Nares Trakooljul, Frieder Hadlich, Eduard Murani, Klaus Wimmers, Siriluck Ponsuksili

**Affiliations:** 1 Research Institute for Farm Animal Biology (FBN), Institute for Genome Biology, Wilhelm-Stahl-Allee 2, 18196 Dummerstorf, Germany; 2 University Rostock, Faculty of Agricultural and Environmental Sciences, 18059 Rostock, Germany

**Keywords:** Genetics, Neuroscience, Computational platforms and environments

## Abstract

Epigenetic dynamics are essential for reconciling stress-induced responses in neuro-endocrine routes between the limbic brain and adrenal gland. CpG methylation associates with the initiation and end of regulatory mechanisms underlying responses critical for survival, and learning. Using Reduced Representation Bisulfite Sequencing, we identified methylation changes of functional relevance for mediating tissue-specific responses in the hippocampus, amygdala, hypothalamus, and adrenal gland in pigs. We identified 4186 differentially methylated CpGs across all tissues, remarkably, enriched for promoters of transcription factors (TFs) of the homeo domain and zinc finger classes. We also detected 5190 differentially methylated regions (DMRs, 748 Mb), with about half unique to a single pairwise. Two structures, the hypothalamus and the hippocampus, displayed 860 unique brain-DMRs, with many linked to regulation of chromatin, nervous development, neurogenesis, and cell-to-cell communication. TF binding motifs for *TFAP2A* and *TFAP2C* are enriched amount DMRs on promoters of other TFs, suggesting their role as master regulators, especially for pathways essential in long-term brain plasticity, memory, and stress responses. Our results reveal sets of TF that, together with CpG methylation, may serve as regulatory switches to modulate limbic brain plasticity and brain-specific molecular genetics in pigs.

## Introduction

An external stimulus that threatens an individual’s integrity (stressors) results in a stress response, an adaptive mechanism to determine, evaluate, and solve challenging situations by defying new coping strategies^1^. The integration between cognitive and regulatory processes from the limbic system, the hypothalamus–pituitary–adrenal axis (HPA axis), and the sympathoadrenal-medullary axis (SAM axis) modulates the initiation, control, and ending of behavioral and emotional responses to stress in the brain^2,3^. This integration is often referred to as the limbic-hypothalamus–pituitary–adrenal-axis (LHPA-axis). In the LHPA-axis, the hippocampus, amygdala, and hypothalamus, the main structures of the limbic system, take control of survival, feeding, breeding, and fight or flight responses^2^. Besides, the hypothalamus, together with the pituitary and the adrenal gland (HPA-axis), modulate the secretion of stress- and anxiety-related hormones^4^. In parallel, via the sympathetic nervous system, the adrenal medulla (SAM-axis), mediates an immediate response to release stress hormones^3^. As result, while the limbic structures interact with the nervous and endocrine pathways in response to exogenous triggers, the adrenal gland becomes the ultimate peripheral organ in both nervous SAM- and endocrine HPA- axes of the stress response. The involvement of these two axes adjusts the homeostatic state in response to stress in the brain and simultaneously influences neurotransmission and synaptic plasticity^5^.

DNA methylation, an integral facet of the epigenome, is critical for defining the brain’s diverse physiology, complex cellular organization, and abundant gene expression under normal, stress, and disease conditions. Internal and external factors influence DNA methylation in the brain, hence having short and long-term effects on the mechanisms that modulate synaptic activity and neuronal plasticity in response to the environment^6,7^. Therefore, characterization of how DNA-methylation variates and influences different tissues from the LHPA-axis may reveal important clues on the regulatory elements that guide tissue specification. It also opens the possibility to assign putative biological roles of candidate regions that may lead to brain functional diversity and plasticity.

Pig’s anatomic, biochemical, physiological, and pathological similarities with humans, make them a valuable model organism for understanding the underlying biology of health and disease, many of clinical relevance^8^. In particular, for some human brain regions, including the hypothalamus, the global expression profile is more similar to that of pigs than mice^8^. Tissue-specific methylation has been demonstrated in pigs, leading towards the understanding of complex traits^9–11^. Furthermore, the mechanisms that underlie animal response to stress are of high interest in the context of animal welfare within food production systems^12^. Our study captures the complexity of variation in DNA CpG methylation of the hippocampus, amygdala, hypothalamus, and adrenal gland, four major limbic, HPA- and SAM- axis structures from German Landrace pigs. We included the adrenal gland, a functionally coupled organ outside the central nervous system, to highlight its contrasts in DNA methylation.

We used reduced representation bisulfite sequencing (RRBS) to generate methylation profiles for 78 tissue samples from 20 animals and implemented pairwise comparisons to estimate the differences across tissues. Together with mRNA expression profiles obtained for the same animals and tissues, we identified and characterized variation across CpG profiles, and explore their putative roles in defying tissue-specific phenotypes. We identified differentially methylated single site CpGs (DMCs) and genomic regions (DMRs) and significantly expanded our knowledge on the epigenetic differences of functional relevance among tissues. Functional annotation of DMCs and DMRs revealed enrichments for differential methylation in promoters of gene regulatory factors (GRFs), of which some are essential gene regulators for brain-specific chromatin remodeling, neurogenesis and cell specialization, and embryonic and endocrine development.

## Results

### Characterizing DNA methylation landscape across pig's LHPA-axis

We build DNA CpG methylation landscapes of four tissues from the LHPA-axis to characterize variation that may be relevant in defining phenotype-specificity for tissues with functional relevance in emotional and behavioral responses. We took samples from the hippocampus, amygdala, hypothalamus, and adrenal gland from the same 20 individuals. We generated about 2.6 billion uniquely mapped 114-bp single-end reads data set. We obtained a mapping efficiency of 79.2 ± 2.6% (Supplementary Table S1). On average, we obtained about 2.8 million single site methylated CpG per sample (SD = 0.77).

**Figure 1:**
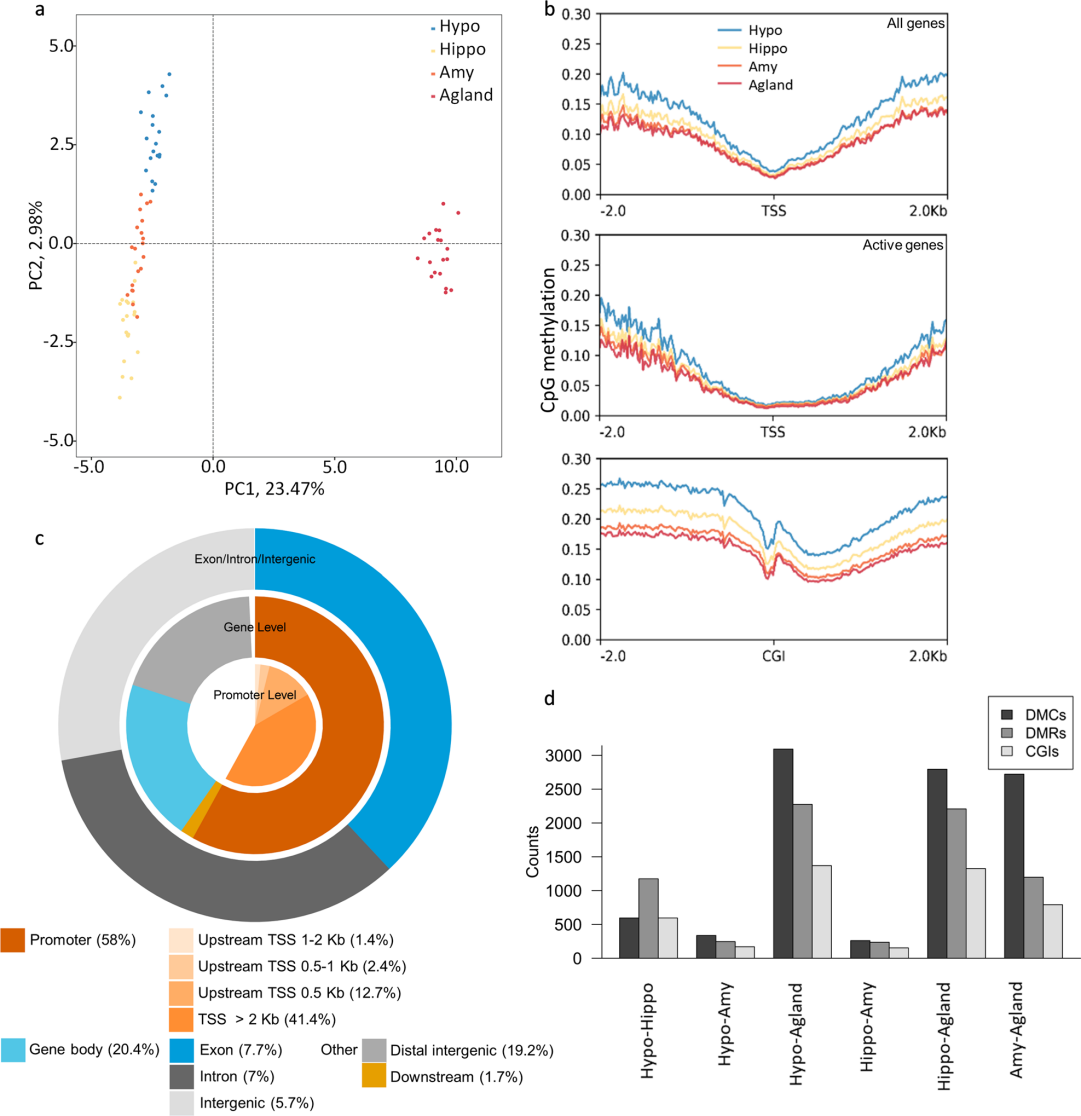
Differentially methylated CpG sites in four tissues: the hypothalamus (Hypo), hippocampus (Hippo), amygdala (Amy), and adrenal gland (Agland) from German Landrace pigs. (**a**) Principal component analysis. (**b**) Profile plot^61^ depicting the methylation rate centered to: TSS of all genes in Sscrofa11.1 (top), TSS of genes with expression values in the mRNA profiling expression data from the same individuals (middle), and to annotated CGIs (bottom). Y-axis shows methylation level as methylation rate per CpG. (**c**) Feature annotation for 60,090 common meCpGs. The percentages for the promoter and gene body are split into sub-features. (**d**) Counts of significantly DMCs and DMRs, and annotated CGIs overlapping with the DMRs detected here.

Principal component analysis of limbic and adrenal CpG DNA methylation landscapes revealed partial segregation of the three brain regions for the first two principal components (Fig. 1a). The hypothalamus displays higher global methylation rate (0.23), followed by the hippocampus (0.19), amygdala (0.17), and adrenal gland (0.15). These differences in methylated CpG (meCpG) rates suggests broad differences across tissues, with the hypothalamus displaying the biggest contrast in the empirical cumulative distribution among all four tissues (Kolmogorov–Smirnov test, D = 0.124, *p* value < 2.2e-16) (Supplementary Figure S1a). Methylation rates around the TSS of all genes suggest similar DNA meCpG rates, with the amygdala and adrenal gland displaying less methylation around the TSS (Fig. 1b, top). These differences are smaller near to the TSS of active genes (Fig. 1b, middle). We additionally observed that differences in meCpG around CpG islands (CGIs) are broader than around TSS. In particular, our results suggest that subsets of CGIs are differentially methylated (hypermethylated) across tissues, while most of the CGIs are still protected from methylation (valley) (Fig. 1b, bottom). These observations suggest that silencing/activation of regions rich in CpG, including CGIs, may be of relevance for modulating tissue-specific DNA regulatory activity through CpG methylation in pig’s LHPA-axis.

### Small differences in single CpGs sites across brain tissues from LHPA-axis

**Figure 2:**
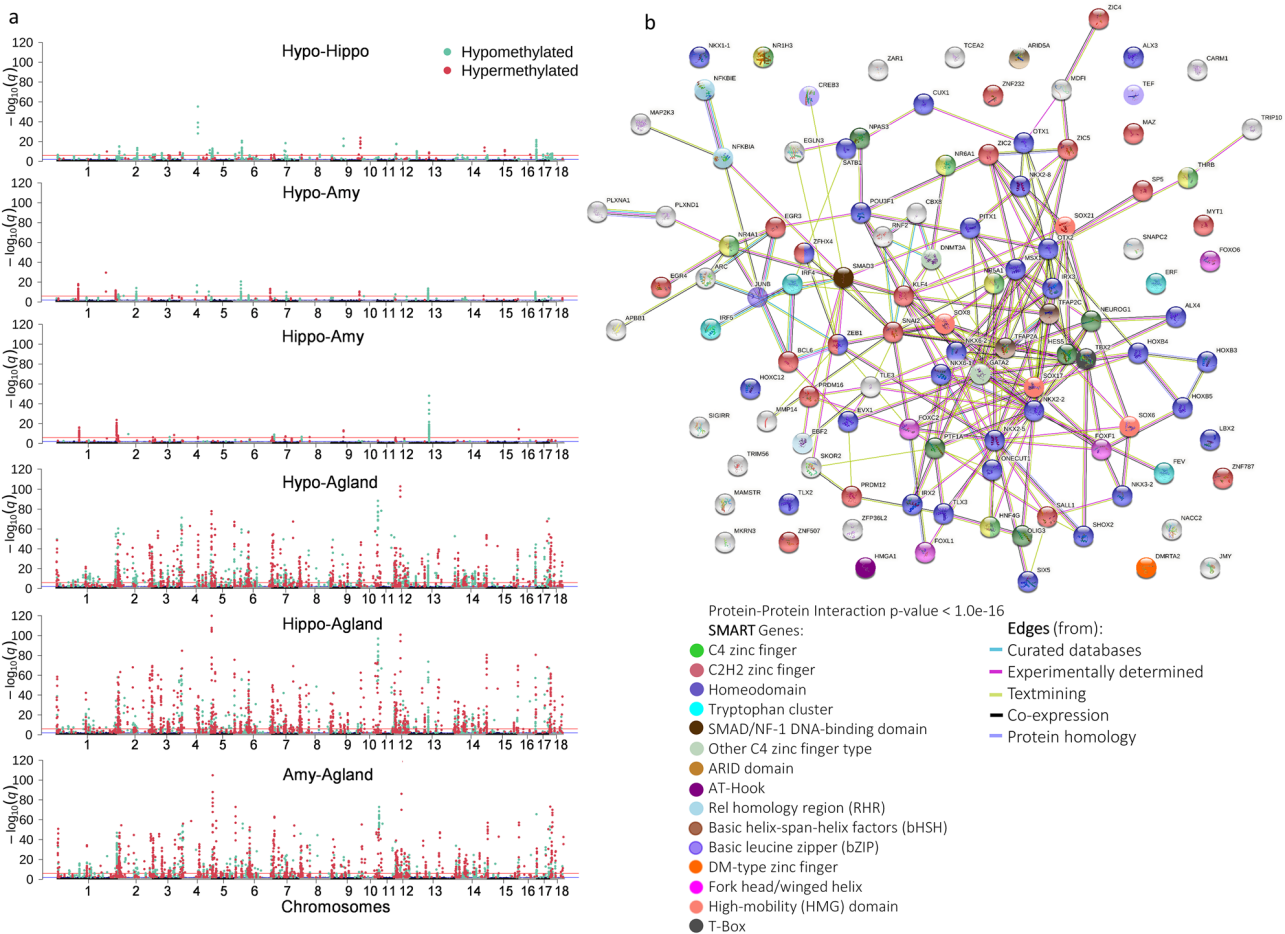
DMCs landscape across Hypo, Hippo, Amy, and Agland. (**a**) Manhattan plots showing pairwise differentially methylated CpGs. Statistical significance set as −log10(q-value) in the y-axis against chromosomes (autosomes) in the x-axis. Significant DMC (q-value < 0.01) are shown in colored dots, hypermethylated (light red), and hypomethylated (light cyan). Genome-wide significance levels are shown horizontal solid lines: −log10(1e-2)(dark blue) and −log10(1e-6)(dark red). (**b**) Protein–protein association STRING network^56^ built using 59 (TF and co-regulators) with at least one DMC among Hypo, Hippo, Amy, and Agland (See Materials and Methods). The full network is shown, disconnected nodes are shown to reveal GRFs whose interaction is not well documented or yet unknown. The network provides discriminatory information between DNA-binding regulators (TF, in multiple colors) and other gene regulators (light grey). Color code shows different classes of DNA-binding TFs based on class, the nodes represent genes, and their color reveals their respective TF class based on Wingender et al. (2018) classification^62^. The color of the edges denotes the type of evidence that supports the interaction between pairs of proteins.

About 99% of all meCpGs sites passed our quality controls, with only a small fraction detected in all tissues (about 2.1%, 60,090/2.84 million) (Supplementary Table S2). About 58% of these meCpGs (34,862/60,090) are near promoters (± 2 kb TSS), with about 41% located 2 kb downstream of the TSS. About 20% of the meCpGs occurred on the gene body, indistinctive of the feature (exon, intron, intergenic 5' or 3' region) (Fig. 1c, Supplementary Table S3). It is apparent that only a small fraction of the meCpGs significantly differ in their methylation rates across all the hypothalamus, hippocampus, amygdala, and adrenal gland (~ 7%, 4186 DMCs/60,090, q-value < 0.01) (Supplementary Table S4). While only a small fraction of DMCs occurs among all three brain tissues (< 1%, n = 11), the hypothalamus displays the larger number of DMCs compared to the amygdala and hippocampus, 8% (n = 337) to 14% (n = 596), respectively (Fig. 1d, Supplementary Table S4). The sets of DMCs among brain tissues suggest a trend towards hypomethylation (hypo) in the hypothalamus: hypothalamus-hippocampus (hyper = 110, hypo = 486), hypothalamus-amygdala (hyper = 123, hypo = 214), and hippocampus-amygdala (hyper = 185, hypo = 75) (Fig. 2a). This difference in methylation is less prominent between the adrenal gland and the brain tissues: hippocampus-adrenal gland (hyper = 1563, hypo = 1232), amygdala-adrenal gland (hyper = 1410, hypo = 1312) and hypothalamus-adrenal gland (hyper = 1320, hypo = 1773) (Fig. 2a).

Annotation to genomic features resulted in a set of 1006 DMCs (24%) occurring in the promoters of 380 genes (Supplementary Table S5). Importantly, we only found a very small, albeit significant, correlation between gene length and the meCpG significance levels (Spearman rank correlation ρ < 0.02, *p* value < 0.07) at pairwise level. This suggests that differences in the gene length probably did not bias our findings and downstream analyses.Among these genes involved in sequence-specific DNA recognition and transcription regulatory activities are enriched (q-value < 0.05) (Supplementary Figure S2, Supplementary Table S6). Similar to their molecular function, enriched biological terms suggest important regulatory roles in cell fate commitment and negative regulation of Notch signaling pathway (q-value < 0.05) (Supplementary Figure S3, Supplementary Table S6). About 29% of these gene set are GRFs (110/380), of which 85 are transcription factors (TFs). Most of these TFs belong to two types of DNA-binging classes: homeodomain (36%, 31/85) and zinc fingers (ZNF) (28%, 24/85) genes (Fig. 2b, Supplementary Table S7). Protein–protein association STRING network reveals a high interconnected set of homeodomain proteins and support robust interactions among them (Fig. 2b). We also detected lack of connectivity in 15 of these GRFs, and speculate the lack of experimental knowledge as likely explanation.

### Large epigenetic differences in DMRs across the LHPA-axis

**Figure 3:**
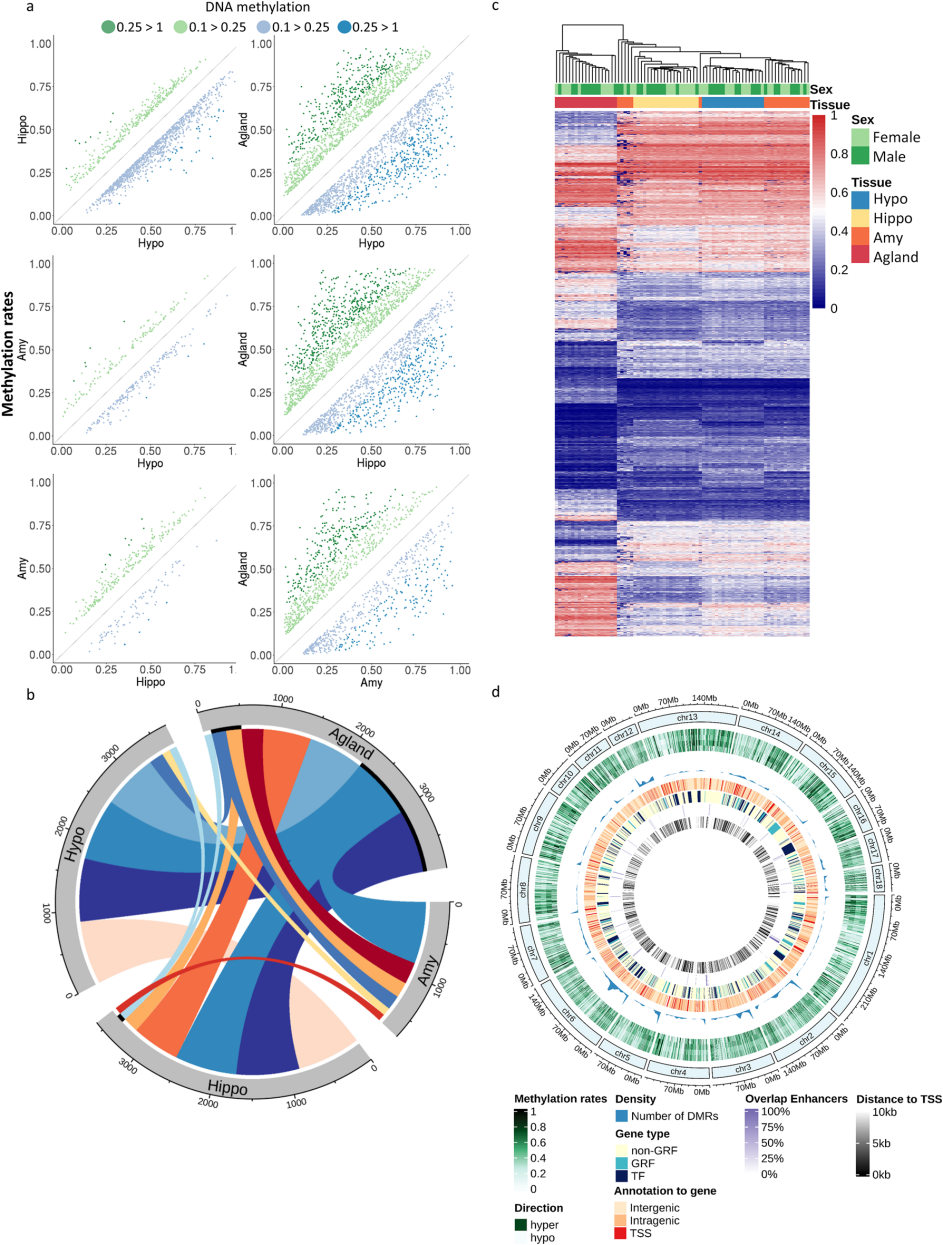
DMRs across Hypo, Hippo, Amy, and Agland. (**a**) Scatter plots of DMR methylation pairwise comparisons (> 10 meCpGs, mean methylation rate > 0.1, and q-value < 0.05). Green and blue indicate an increase or decrease in methylation compared across tissues, respectively (> = 0.1, light, >  = 0.25, dark). (**b**) Circos plot^63^ displaying 85% (4959/5787) of the total overlapping DMRs detected across pairwise tissue comparisons (Supplementary Table S9). Thick inner black line indicate overlaps of non-unique to pair DMRs. Only overlaps greater than 95 are shown. (**c**) Hierarchical clustering of samples based on DMR’s mean methylation rates per sample. (**d**) Circos plot^63^ displaying DMR's methylation rates and annotation across chromosomes. From outside to inside of the plot: mean methylation rates for Hypo, Hippo, Amy, and Agland (green, hypomethylated: light, hypermethylated: dark), the genomic density of regions (fraction of DMRs per chromosome), annotation to gene, type of gene (TF, GRF, other), annotation to enhancers in pigs^13^, and distance to TSS.

We identified 5190 DMRs across tissues covering 748 Mb (Supplementary Table S8). These DMRs are on average 144 bp long and have up to 113 meCpGs. Between 51% up to 69% of DMRs overlap with annotated CGIs (Fig. 1d), indicating an over-representation among CGIs (approximate permutation test, *p* value < 0.001). Overlaps between DMCs and DMRs suggest 10% of the DMRs (500/5190) overlap with 34% of the DMCs (1414/5190). While the majority of the DMRs (68%, 3532/5190) occur between the brain-LHPA-axis tissues and adrenal (Fig. 3a, Supplementary Table S9), the remaining 32% (1658/5190) are unique, defined as DMR in one pairwise or among one tissue versus the rest, among brain-LHPA-axis tissues. The set of unique DMRs is about half 53.1% of the total set (2758/5190) (Fig. 3b). We also detected that a majority of brain-DMRs occur between the hypothalamus and the hippocampus. Using the mean methylation rates from all DMRs, aside from five samples from the amygdala (25%), we were able to discriminate brain tissues into three clear groups, with brain-LHPA-axis tissues grouped apart from the adrenal gland (Fig. 3c). Conversely, hierarchical and dimensional reduction analysis of DMRs suggest that the sex of the animal has not influence in the methylation levels detected in this set of DMRs (Fig. 3c and Supplementary Figure S1b, respectively).

CpG methylation across DMRs, its distribution, and annotation show that pig's chromosomes six (chr6), two (chr2), three (chr3), and 12 (chr12) have a higher density of DMRs, and explain about 40.5% of the total DMRs (2102/5190) (Fig. 3d, methylation and density tracks). Annotation of DMRs to genomic features revealed that about 45% are in promoters (10%, 543/5190, 338 genes, Supplementary Table S10) and intragenic regions (35%, 1800/5190). The remaining 55% of DMRs (2850/5190) are intergenic (Fig. 3d, Supplementary Table S10). Importantly, there was no correlation between the gene length and the significance level for DMRs (Spearman rank correlation ρ < 0.0003, *p* value > 0.05,). Annotation of DMRs to enhancers from multiple tissues and breeds from pigs^13^ rendered only a small fraction (0.01%) of overlap (Fig. 3d). About 26% of the total DMRs are close to GRF (promoters or intragenic), 16% of them are TFs (Fig. 3d). Among genes with DMRs in promoters, 41% (137/338) display significant changes in expression in at least one pairwise (q-value < 0.05) (Supplementary Table S10). Taken together, our results point towards regions where DNA methylation may be imperative for silencing-activating promoters, especially for a set of GRFs that may influence tissue- and organ-specific gene regulatory pathways in pigs’ LHPA-axis.

### Hippocampus and hypothalamus display remarkable epigenetic differences in genes critical for neuronal development and brain plasticity

Hierarchical clustering of samples based on the mean methylation rates from DMRs suggests that the hippocampus clusters apart from the hypothalamus and amygdala (Fig. 3c). While the amygdala displays the smallest number of unique DMRs among brain-LHPA-axis tissues (3.5–4%), unique DMRs between the hippocampus and hypothalamus displayed up to nine times more. These unique DMRs represent about 31% of the total unique DMRs among LHPA-axis’ tissues (860/2758), and it is comparable in number to those detected between brain-LHPA-axis and the adrenal gland (30%, 839/2758) (Fig. 3b, Supplementary Table S9). Compared to hippocampus, the methylation rates of these 860 unique DMRs indicate a decrease in the hypothalamus (Supplementary Figure S5). Annotation to genes revealed a small fraction of these DMRs (6%, 52/1175) occur in the promoters of 48 genes (Supplementary Table S10). Expression profile contrasts suggest that at least 21% (10/48) of these genes are DE between hippocampus and hypothalamus (q-value < 0.05): *ADARB1, CDH22, CFL1, CH25H, EFNA1, POLDIP3, SS18L1, TNFAIP8L2, UBE2S,* and *ZNF385A*. We detected that 38% of the genes with DMRs in promoters (18/48) have unique DMRs to the pair hippocampus-hypothalamus: *C11orf42, CDH22, CEND1, CFL1, CH25H, COL9A3, CPED1, FOXJ1, HIC1, IL17B, KBTBD11, LHX2, PHLDA2, PKNOX2, POLDIP3, SS18L1, TNNT3,* and *ZNHIT2*. Only five genes of these 18 genes are DE between both tissues: *CDH22, CFL1, CH25H, POLDIP3,* and *SS18L1*.

Ontological enrichments for these 48 genes indicate an overrepresentation of gene regulatory processes such as sequence-specific DNA-binding, transcription factor activity, regulatory region nucleic acid binding, among others (q-value < 0.01) (Supplementary Figure S6a). These analyses derived into the regulation of specific biological processes for neuronal precursor cells proliferation, T-cell activation, Notch signaling, lymphocyte, and leukocyte, as well as organ and nervous system morphogenesis and development, learning, and memory (q-value < 0.01, Supplementary Figure S6b). Further characterization revealed 17 genes that encode GRF proteins: 13 TF (*FOXF1, FOXJ1, GRHL2, HIC1, IRX1, IRX3, LHX2, MSX1, PAX1, PKNOX2, SOX6, SP5,* and *ZNF385A*) and four co-regulators (*ANKRD2, RGS14, SLC4A10,* and *SS18L1*). Intriguingly, two of these 17 GRFs are critical for neuro-physiological tissue-specific processes: *SS18L1* and *LHX2*^14,15^. The Subunit of BAF Chromatin Remodeling Complex (*SS18L1*), as well known as CREST, has a unique DMR to the pair located 1664 bp upstream the TSS (40 bp in length, q-value < 0.0005). Similar to *SS18L1*, *LHX2* harbors a DMR unique to the pair on its promoter (249 bp in length, q-value < 5.21e-13) (Supplementary Figure S7a). Although *SS18L1* expression is ubiquitous (Genotype Tissue Expression project, GTEx)^16^, we detected significantly higher expression in the hypothalamus (q-value < 0.05). Conversely, *LHX2* is mostly expressed in the brain (GTEx)^8,16^, being up to 6.5 times higher in the hippocampus and amygdala than the hypothalamus (Protein Pig Atlas, PPA-pig data)^8^ (Supplementary Figure S7b and c). Taken together, we suggest that apart from SS18L1 and LHX2, the remnant 15 GRFs may also play critical neuro-physiological regulatory roles in pig’s LHPA-axis.

Among non-GRFs, we also detected two genes with unique DMRs in their promoters and that are critical for the limbic brain: Cadherin 22 (*CDH22*) and cell cycle exit and neuronal differentiation protein one (*CEND1*). *CDH22* is critical in the morphogenesis, development, and maintenance of brain tissues^17–19^. Our analysis revealed two DMRs in the promoter of *CDH22*, one overlapping the TSS (227 bp in length, q-value < 2.13e-70) and one 138 bp downstream the TSS (90 bp in length, q-value < 1.99e-17, Supplementary Figure S8a). Both DMRs display significantly higher methylation in the hypothalamus than in the hippocampus. These two DMRs overlap with an annotated promoter CGI for Sscrofa11.1 (Supplementary Figure S8a). Similar to *CDH22*, *CEND1*, a neuron-specific gene with critical roles during persistent neurogenesis in the hippocampal dentate gyrus^19^, harbors a DMR 695 bp downstream of its TSS (152 bp in length, q-value < 8.27e-06) (Supplementary Figure S8). Although *CDH22* and *CEND1* expression is ubiquitous^16^, the order of magnitude drastically increases for brain tissues. In pig’s brain, *CEND1*’s expression is about 1.7 times higher in the hippocampus than in amygdala and hypothalamus (GTEx and PPA-pig data, Supplementary Figure S8c and d). Altogether, our results discriminated TF, co-regulatory, and non-GRFs genes where changes in DNA activity may differentially influence critical tissue-specific neuro-regulatory and -physiological processes in hippocampus and hypothalamus.

### Amygdala displays greater methylation similarities with other brain-LHPA-axis

The amygdala, the primary center of the response to fear in vertebrates, shows about four times less DMRs across brain tissue comparisons (Fig. 3a). Despite the smaller number of DMRs detected between the amygdala versus the hypothalamus (n = 246) and the hippocampus (n = 237), these sufficed to classify the majority of the samples into one distinctive cluster (Fig. 3c). Most of the samples from the amygdala clustered together with the hypothalamus, suggesting a higher similarity in their methylation rates. Out of these two sets of DMRs, 40% to 44% are unique to pairwise comparisons with the hypothalamus (109/246) and hippocampus (95/237) (Fig. 3c). Annotation to genomic features highlighted 36 of these DMRs in the promoters of 27 genes, where more than half encode for GRFS: TFs (*FOXF1, FOXI2, MN1, IRX1, IRX3, MSX1, NEUROG1, PAX1, PRDM12, SNAI2, SOX6, SP5,* and *ZNF385A*), and co-regulators (*ANKRD2, SKOR2,* and *SLC4A10*). Among non-GRF, we detected 11 coding genes: *CUEDC1, TPRN, LOXL1, CDH22, FZD8, DLEU7, TNNT3, IFITM5, GPR35, INSYN1,* and *RIPK4*.

The disjunctive union of genes with DMRs between the hypothalamus, hippocampus, and the amygdala, revealed differentially methylated promoters of 11 genes: six TFs (*FOXI2, NEUROG1, MN1, PRDM12, SKOR2,* and *SNAI2*) and five non-GRFs (*INSYN1, RIPK4, LOXL1, FZD8*, and *DLEU7*). Three out of these non-GRFs genes encode cell-receptor proteins: *INSYN1, RIPK4,* and *FZD8*. We detected five unique DMRs in promoters of two genes, four located in the promoter of *SKOR2* and one on *DLEU7*. SKI Family Transcriptional Corepressor 2 *SKOR2* is essential for Purkinje cells’ morphogenesis, migration, and differentiation, and may take critical roles in the RAR-related orphan receptor alpha (RORα) pathway in brain^20,21^. Four DMRs located between 480 and 1868 bp downstream the TSS of *SKOR2* partially overlap with a CGI (chr1, CpG 268, 2590 bp in length, Supplementary Figure S9a). Further exploration revealed that *SKOR2* is only expressed, and at a very low level, in brain tissues, and testis from humans (GTEx data, Supplementary Figure S9b). In pigs, *SKOR2* is expressed in the hypothalamus, cerebellum, basal ganglia, pons and medulla, spinal cord, and in the retina, but not in the amygdala nor hippocampus (PPA-pig data, Supplementary Figure S9c). Similarly, Deleted In Lymphocytic Leukemia 7 (*DLEU7*) gene functions as a potent NF-kappaB inhibitor, a signaling pathway also implicated in processes synaptic plasticity and memory^22,23^.

Ontology enrichment analysis of genes with DMRs in the promoter between the amygdala versus the hypothalamus and hippocampus highlighted biological processes of relevance in cell–cell surface receptor signaling and peptidyl lysine modification biological processes (only in amygdala-hypothalamus pairwise) (Supplementary Figure S6b). Among those enriched genes, the Inhibitory Synaptic Factor one (*INSYN1*) is a critical protein for the dystroglycan complex in the GABAergic synapses, excitatory/inhibitory balance in brain^24^. *INSYN1*’s promoter has five DRMs, with the amygdala and hippocampus exhibiting higher methylation across tissues (Supplementary Figure S10a). *INSYN1* expression is ubiquitous, although much higher in brain (GTEx data, Supplementary Figure S10b). *INSYN1* is expressed between 2.3 to 2.5 times more in the amygdala and hippocampus than in the hypothalamus in pigs (PPA-pig data, Supplementary Figure S10d). Intriguingly, these five DMRs also overlap with the promoter of the copper ion binding and oxidoreductase activity gene *LOXL1*. *LOXL1* has significantly higher expression, up to 2.4 times more, in the hypothalamus than in amygdala or hippocampus (q-value < 5.49e-06, Supplementary Figure S10c). This suggests that the machinery controlling CpG methylation in the promoters of *LOXL1* and *INSYN1* may exert differential effects on their expression levels.

### DMRs highlight instructors of regulatory changes between the brain and adrenal gland

Compared to the adrenal gland, the hippocampus and hypothalamus exhibit numerous differences in DMRs, ranging from 62% (2208/3535) to 64% (2275/3535), respectively. This number is about 53% lower in the amygdala-adrenal gland pairwise (1198 DMRs). Only about 9.5% of these DMRs (337/3535) rest in the promoters of 317 protein-coding genes (Supplementary Table S11). We detected an enrichment of genes with molecular functions for sequence-specific DNA recognition, regulatory, and enhancing activities (q-value < 0.01, Supplementary Figure S6a). Further inspection of the functional roles of these 317 protein-coding genes pinpointed 69 GRFs that carry at least one DMR in their promoters, of which 41 encode for TFs (Supplementary Table S12). We also detected 66% of these TFs (27/41) belong to four TF classes: C2H2-ZNF (n = 10), homeodomain (n = 9), Forkhead/winged-helix (n = 4) and bHLH (n = 4). Aside from the enrichment for regulatory activities, we also identified an enrichment of genes for biological processes such as cell development, specialization, and cell-to-cell communication, and embryonic, endocrine, and tissue morphogenesis and development (Supplementary Figure S6b, Supplementary Table S13). This set of GRF genes may rule functional tissue specific differences between brain-LHPA and differential, and subsequently the initiation or repression of regulatory pathways across pigs LHPA-axis.

### Transcription factor binding sites of master regulators enriched among DMRs in the brain

**Figure 4:**
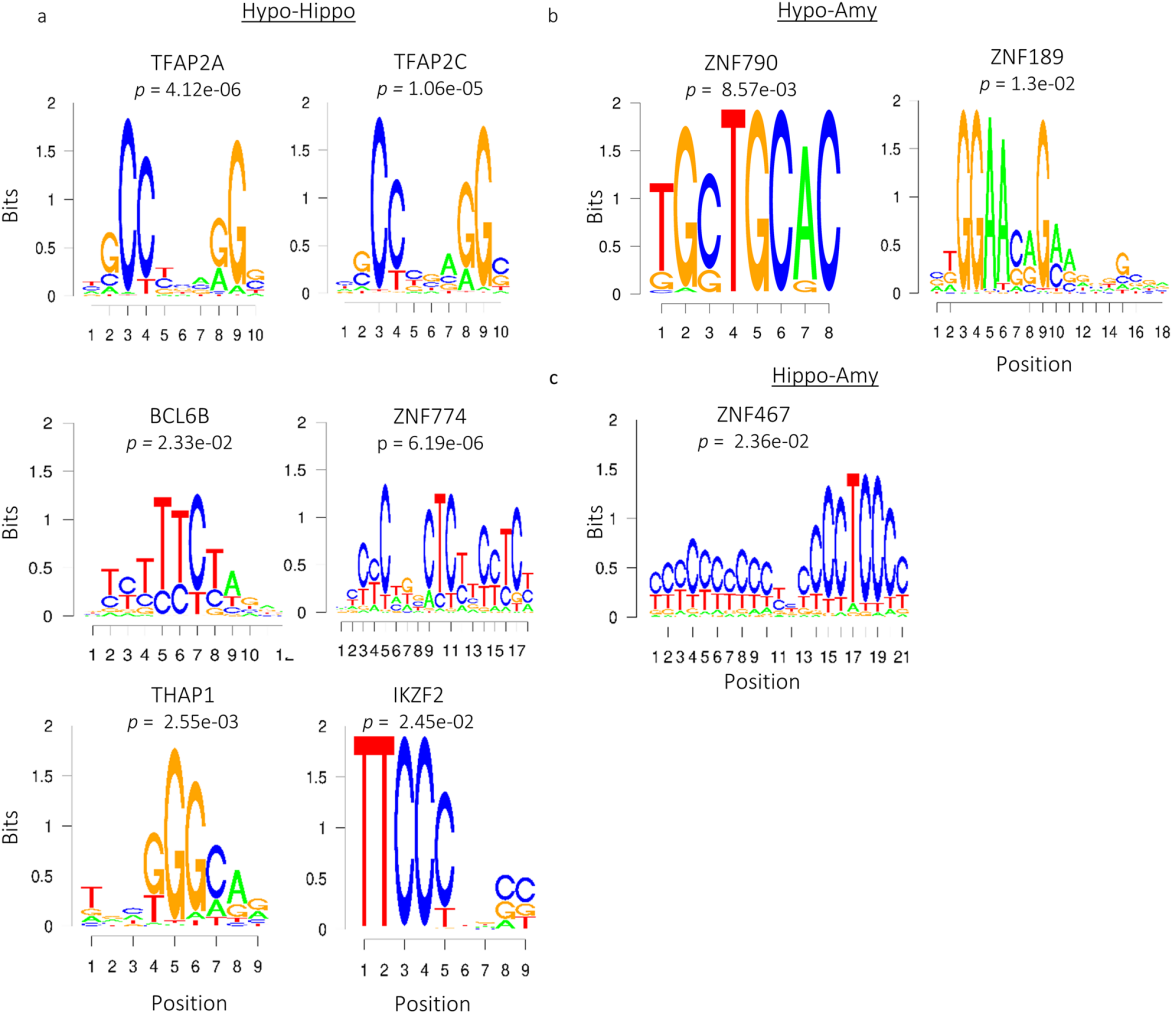
TFBM enriched within DMRs in promoters of genes among brain pairwise comparisons. (**a**) Hypothalamus-hippocampus. (**b**) Hypothalamus-amygdala. (**c**) Hippocampus-amygdala.

By exploring the co-occurrence of TFBS within DMRs in the promoters of genes (± 2 kb around the TSS), we identified binding sites for TFs that may exert control on gene expression of genes with relevance for LHPA-axis' tissues. Enrichment analysis of TFBS motifs on DMRs from promoters of 48 genes revealed TF binding motifs (TFBM) where six TFs putatively bind about 50% of these regions: TFAP2A, TFAP2C, ZNF774, THAP1, BCL6B, and IKZF2 (Fig. 4, Supplementary Table S14). Two AP-2 proteins, Transcription Factors AP-2 (TFAP2A) and 2C (TFAP2C*)*, may bind and regulate between 58% (28/48) to 77% (37/48) of these promoters (Bonferroni corrected *p* value < 1.06e-06). Interestingly, six putative target genes of these two AP-2 proteins encode for six TFs (*FOXJ1, GRHL2, IRX1, LHX2, SP5*, and *ZNF385A*) and three GRFs (*ANKRD2, RGS14,* and *SS18L1*). We additionally detected enrichments of TFBM for ZNF774, THAP1, IKZF2, and BCL6B (Fig. 4) in promoters where TFAP2A and TFAP2C may also bind. This suggests them as co-regulatory partners for these two AP-2 proteins (Supplementary Table S15).

We also identified three enriched TFBM for three ZNF proteins among brain tissues: ZNF790, ZNF189, and ZNF467 (Bonferroni corrected *p* value < 0.013, Supplementary Table S14). Among their putative target genes, ZNF790 may bind in the promoter of eight genes (*CUEDC1, DLEU7, FZD8, INSYN1, IRX1, LOXL1, MN1,* and *SKOR2*), ZNF189 on eight (*DLEU7, FOXI2, GPR35, IRX3, MN1, NEUROG1, SNAI2,* and *SP5*), and ZNF467 on seven (A*NKRD2, MN1, MSX1, PRDM12, SOX6, TPRN,* and *ZNF3*85A). Our data suggest that these three ZNF proteins interact with the promoter of *MN1*, a transcriptional co-regulator critical for brain development and cognitive functioning^25,26^. We additionally found that *ZNF790* and *ZNF189* may also bind one DMR located in the promoter of *DLEU7*. *DLEU7* encodes an important protein implicated in processes synaptic plasticity and memory^22,23^.

## Discussion

The LHPA-axis integrates brain and endocrine structures that support fundamental biological functions such as emotions, behavior, long-term memory, olfaction, and central stress-response. Our study constitutes the largest CpG DNA epigenome-mapping across the hippocampus, amygdala, hypothalamus, and adrenal gland from pigs. Our focus on these four tissues is relevant for understanding tissue-specific epigenetic regulation of relevance for neuro-developmental, endocrine, physiological, and behavioral responses to stress. We performed the first analysis of differential methylation among limbic tissues in pigs (hippocampus, amygdala, and hypothalamus), identifying DMC and DMRs located in neuro-specific gene regulators. We used the adrenal gland, an endocrine organ highly integrated with the LHPA-axis, to highlight its differences with limbic brain DNA methylation. We identified about 2.8 million meCpG, with 60,090 detected in all 78 tissue-samples. Our results suggests a small fraction of meCpGs significantly changes their methylation rates across the four tissues (n = 4186 DMCs). About 24% (n = 1006) of DMCs lay in promoters of genes encoding for sequence-DNA specific binding proteins (TFs) and other co-regulatory genes. Our results pinpoint CpG methylation sites with putative roles in fine-tuning the expression of neuro-regulatory proteins from the LHPA-axis like cell specialization and communication, embryonic and endocrine development, tissue morphogenesis, and neuro-specific cellular processes.

Analysis of DMRs significantly expanded our knowledge of differentially methylated DNA across pig's limbic and adrenal structures. We identified 5190 covering 748 Mb, of which up to 53% correspond to unique to the pair DMRs. Compared to single site analyses, our results suggest that genomic regions displaying significantly different methylation statuses across tissues have larger effects in silencing-activating the DNA segments of functional relevance across pig’s LHPA-axis. Annotation of DMRs suggests great differences in chromosomes chr3 and chr12, autosomes that were previously associated with molecular and phenotypic variation in coping behavior-associated traits in pigs^10^.

DMRs between the hippocampus and the hypothalamus locate on genes with distinctive functional roles in LHPA-axis. For instance, DMRs in promoters of 17 TF essential for modulating brain-specific regulatory mechanisms. Their functional association, together with our methylation data, suggests that variation in DNA activation of these promoters may influence the expression of gene regulatory proteins critical for neuronal precursor cells. Neuronal precursor cells are the basic units with critical roles for brain development, normal neuroplasticity, learning, and memory in the hippocampus. Among genes with DMRs between the hippocampus, amygdala, and hypothalamus, four stick out by the significant differences in the methylation of their promoters, expression profiles, and functional characterization: *SS18L1, LHX2, CEND1,* and *CDH22*. *SS18L1* encodes the CREST protein, an essential subunit of a neuron-specific chromatin-remodeling complex for long-term effects on neuro-functional processes^14,27^. Calcium-dependent gene expression and signaling by CREST's prolonged effect critically influences dendritic growth and long-term brain plasticity, neurogenesis, learning, and memory processes in hippocampus^28^. Similarly, *LHX2* encodes a protein with critical roles in astrogliogenesis and promotes neurogenesis in the developing hippocampus^15^. LHX2, together with CEND1, are instructive TFs that lead the regulation of neuronal precursor marker genes and promote temporarily regulated neuronal differentiation^19,29^, a remarkable feature found in adult hippocampal neurogenesis in response to the environment. Similarly, the *CDH22* gene encodes a cell adhesion transmembranal protein critical in guidance and recognition for developing axons during the elongation. Our results suggest that activation/inactivation of the promoters of *SS18L1*, *LHX2*, *CEND1,* and CDH22 throughout CpG methylation may be important for fine-tuning neuronal progenitors division, neuronal proliferation, and differentiation in pig’s brain-LHPA-axis, especially in the hippocampus.

Our TFBS motif enrichment analysis also revealed that TFAP2A and TFAP2C, two TF master regulators in lipid biogenesis^30^, may play similar key roles in limbic brain. These two TFs may enhance the expression of at least other seven regulatory genes critical for multiple neurodevelopmental and neurophysiological processes: *FOXJ1, LHX2, ZNF385A, SS18L1, GRHL2, SP5,* and *EFNA1*. FOXJ1 is involved in cell fate determination of neurons^31^, LHX2 is essential during neurogenesis in the hippocampus^15^ and forebrain development^32^, ZNF385A targets *ITPR1* mRNA to dendrites in Purkinje cells^14^, SS18L1 acts as essential subunit calcium-responsive transactivator of neuron-specific chromatin-remodeling complex^14^, GRHL2 regulate pathways involved in motor-coordination and inhibition of anxiety^33^, SP5 regulates myelin basic protein (MBP) gene expression in mature oligodendrocytes^34^, and EFNA1 has major roles in cell migration, repulsion, and adhesion of neuronal cells^14^. Further studies on TFAP2A and TFAP2C are yet necessary to validate their roles as "master" regulators in brain plasticity, memory, and stress-response.

The smaller number of DMRs in promoters between the amygdala and the hippocampus may relate to their physiological association in the limbic brain. Intriguingly, clustering of the samples using mean methylation rates for DMRs revealed five samples (25%) from the amygdala clustered outside the branch grouping all brain structures. We speculate that the variation in the methylation rates observed for DMRs in these five samples could correspond with an over-representation of one or more subregions from the amygdala (laterobasal, centromedial, and superficial nuclei^35,36^), or with a specific phenotype-environment interaction for us yet unknown.

Although methylation profiles, and subsequent gene expression, variate across the lifespan and environmental conditions, these data uncovered genomic locations that could be critical for differentially activating or silencing many gene regulatory factors that are imperative for fine-tuning the expression of other genes. The differential methylation observed in gene regulators, many homeodomain and C2H2-ZNF among them, pinpoint towards differences that are pivotal for neurogenesis, brain development, physiology, and pathology^37–41^. We postulate these sets of TFs and coregulators for the experimental follow-up to expand our knowledge on their regulatory roles in early developing pigs, and during brain tissue specification and physiological diversification, for instance, within the context of plasticity, memory, and stress-response in farmed animals.

## Conclusions

RRBS technique uses specific restriction enzymes to enrich and capture CpG-rich methylated regions, thus showing bias towards CpG rich sites, commonly studied dinucleotides often found in clusters such as DMRs and CGIs. RRBS data represents only a part of the cytosines in the genome of vertebrates and has a bias towards better-annotated regions^42^, leaving extensive areas of methylated DNA containing other types of DNA regions of biological relevance, for instance, repetitive sequences, short and long interspersed nuclear elements uncovered. Markedly, DNA methylation also occurs in non-CpG sequence contexts (CHH and CHG), highlighting the relevance of implementing other methods to analyze a broader repertoire of methylated cytosines. In the context of studying methylation in brain tissues, for instance, between 14 to 40% of methylation marks correspond to elevated levels of hydroxymethylation^43,44^. In addition, the use of other molecular tools, for instance, RNA-seq, will expand the possibilities to explore the putative roles of DMCs and DMRs occurring within the structure of genes. Such DMC and DMRs may play critical roles in transcriptional processes such as silencing alternative transcriptional start sites, transcriptional elongation, and isoform expression. Consequently, studies using other DNA methylation techniques that cover larger areas of the genome, coupled with a multi Omics strategy, will help expand our knowledge of the methylome from the LHPA-axis in pigs.

## Materials and methods

### Ethical statement

Animal care and tissue collection followed the guidelines of the German Law of Animal Protection. The experimental protocol was approved by the animal care committee at the research Institute for Farm Animal Biology as well as by the State Mecklenburg-Western Pomerania (Landesamt für Landwirtschaft, Lebensmittelsicherheit und Fischerei; LALLF M-V/TSD/7221.3-2.1-020/09). The experimental protocol followed the approved guidelines for safeguarding good scientific practice at the institutions of the Leibniz Association. Measures were taken to minimize the pain and discomfort, and were in agreement with the guidelines laid down by the European Communities Council Directive of 24 November 1986 (86/609/EEC). The authors confirms that the study is reported in accordance with ARRIVE guidelines.

### Sample collection

The 20 animals used, 10 females and 10 castrated males (sex balanced), are a subset of 294 German Landrace pigs formerly studied for identifying traits of relevance for understanding animal coping behavior^10^. Tissue samples from the hippocampus, amygdala, hypothalamus, and adrenal gland from the same animals were collected and provided by the research Institute for Farm Animal Biology (FBN, Dummerstorf, Germany). Briefly, the animals used here were sampled according to the slaughter routine at an average age of 157 ± 10 days at a slaughter weight of 85 ± 6.4 kg. Pigs were weighted and slaughtered by electronarcosis followed by exsanguination. Tissue samples for DNA isolation were taken immediately after exsanguination. A stereotaxic atlas of the porcine brain was used as anatomical reference guide^45^. The hypothalamic area, including the paraventricular nucleus, was surgically removed from the left and right hemispheres soon after harvesting the brains. Similarly, the middle portions of both adrenal glands, the amygdala and its nuclei, and the hippocampus were localized and excised. Tissues from all four tissues were snap-frozen in liquid nitrogen and stored at -80 °C.

#### DNA isolation, library construction, bisulfite sequencing

Genomic DNA was extracted from samples using a DNeasy kit (Qiagen) according to the manufacturer’s recommendation including proteinase K digestion and RNase A treatment to obtain high purity DNA. We used RRBS to measure genome-wide methylation profiles at a single nucleotide level^46^. We used double enzyme (MspI and TaqαI) digestion and increased selected-fragment size. As we previously reported^47^, the RRBS libraries were built using 2 µg of DNA with a 1% spike-in control (unmethylated cl857 Sam7 Lambda DNA, Promega) and digested with MspI and Taq αI. Several samples were multiplexed per sequencing lane using the Illumina TruSeq DNA library preparation kit. Once purified, the digested DNA fragments were end-repaired, A-tailed, and ligated to C-methylated adapters using a TruSeq Nano DNA Sample Preparation kit (Illumina) according to the manufacturer’s recommendations. Next, adapter-ligated DNA fragments were size-selected on 2% low-range ultra-agarose gels to obtain inserts of 40–240 base pairs (bp). The purified DNA library was subjected to bisulfite conversion using an EpiTect Bisulfite kit (Qiagen). Minimal PCR amplification of the library was performed using a PfuTurbo Cx Hotstart DNA Polymerase kit (Stratagene). The quality of RRBS libraries was assessed using an Agilent DNA 1000 kit (Agilent Technologies). In total, we produced 78 RRBS libraries: hippocampus (n = 20), amygdala (n = 20), hypothalamus (n = 19), and adrenal gland (n = 19). Next generation sequencing of the RRBS libraries was performed on an Illumina HiSeq2500 at the FBN, Dummerstorf, Germany.

#### RRBS mapping and methylation call

We pre-processed the FASTQ files to control for base-calling accuracy (Phred quality score, Q > 20), minimum read length (30 bp), and absence of N calls in the read's sequence. We removed two synthetically added blunt-end bases from each 5' and 3' ends. Processed reads were aligned to the *Sus scrofa* genome (assembly Sscrofa11.1)^48^ using the default options from bwa-meth (v0.2.2)^49^. We filtered out sequence alignments for not primarily aligned reads, supplementary, that failed platform quality check, or had a minimum MAPQ score of >= 10. We marked duplicates (samtools)^50^ and extracted per-base metrics using MethylDackel (v0.5.0; https://github.com/dpryan79/MethylDackel).

#### Differentially methylated sites and regions

Single site methylated CpGs (meCpG) were filtered based on their coverage (> = 10) followed up by coverage normalization between samples using methylKit (v1.12.0)^51^. We removed meCpG positions with known C > T mutations that could represent methylation calls inferred from true Ts. Here we used the variant calling file from the Sscrofa11.1 assembly and a high confidence SNP call from genome-wide SNP chip-array data from a subset of 20 of the samples^10^. We used the logistic regression-based model to test for different meCpG proportions, included the sex of the animal as influencing factor, and excluded sex chromosomes from our analysis (MethylKit pipeline). We considered meCpGs with coverage in all samples for downstream analysis. We defined as differentially methylated meCpG (DMCs) those with a q-value < 0.01 (false discovery rate, FDR). We further characterized the different epigenetic states by identifying genomic regions displaying different methylation statuses among two or more tissues (differentially methylated regions, DMRs) using the binary segmentation algorithm and the de-novo default settings using metilene^52^ across pairwise: hypothalamus-hippocampus, hypothalamus-amygdala, hippocampus-amygdala, hypothalamus-adrenal gland, hippocampus-adrenal gland, and amygdala-adrenal gland. We defined as significantly different all those DMRs with at least 10 meCpGs, a minimum absolute mean methylation rates difference of 0.1, and a q-value < 0.05 (Mann–Whitney U test). Sscrofa11.1's genomic coordinates for annotated CpG islands (CGIs) were retrieved from the University of California Santa Cruz (UCSC) genome browser.

#### Annotation and functional analysis

Feature annotation was performed using ChIPpeakAnno (v3.24.1)^53^ and the Ensembl Sscrofa11.1 gene build v102^54^. We defined ± 2 kb from the Transcription Start Site (TSS) as the promoter region. Gene ontology (GO) and pathway (Kyoto encyclopedia of genes and genomes, KEGG) enrichment analysis were used to characterize putative roles of the annotated DMCs and DMRs (FDR < 0.05). We used the hypergeometric test (phyper) and those genes having meCpG annotated inside their structure were considered as background set. To further characterize if genes with DMRs in the promoter region encode for proteins that regulate the expression of other genes (GRFs), we used Ensembl BioMart to obtain one to one orthologs between Sscrofa11.1^54^ and the human catalog of GRFs^55^. In total, we were able to identify 2585 GRFs, of which 1132 are DNA-binding transcription factors. We additionally used STRING to construct functional annotation networks of protein-coding genes^56^ with DMC and DMR in promoters. STRING networks were built using all the available evidence from known interactions, predicted interactions, text mining, co-expression, and homology. The minimum interaction score was 0.400. The max number of interactors to show was set to "None”.

#### Transcription factor binding motifs enrichment in differentially methylated regions

To identify putative transcription factor binding sites (TFBS) and motifs (TFBM) located in DMRs, we used the Analysis of Motif Enrichment tool (AME)^57^. We used TFBS profiles available for *Sus scrofa* in the catalog of Inferred Sequence Binding Preferences (CIS-BP)^58^ and the non-redundant motifs from vertebrates from the JASPAR database^59^. We compared Sscrofa11.1 DNA sequences (primary) to sequences created by shuffling the letters in each primary sequence (control). The background and shuffled sequences, both used for estimating the significance levels, resulted from converting a frequency matrix to a log-odds score and control sequences as described in AME package, respectively. Once detected, we used putative enriched TFBS together with the DMRs annotation to identify putative target genes.

#### Gene expression data sets

We used the snowball microarray data (Affymetrix) genes to build up the expression profiles of the same 78 tissue samples used in the RRBS experiment. We used the Robust Multichip Average (RMA) normalization followed by differential expression analysis using the Limma package^60^. We measured the differential expression of multiple interactions using all possible contrasts (six possible as described above), considered sex as influencing factor, and the moderated contrast t-test for each gene. We controlled the proportions of false positives using a FDR set as q-value < 0.05. For comparative purposes, we sourced the Genotype-Tissue Expression database (GTEx)^16^ and the pig's brain expression atlas (PPA-pig)^8^.

## Supplementary Information

Supplementary Tables.

Supplementary Figures.

## Data Availability

The RRBS data set generated is in the ArrayExpress–functional genomics database under the accession E-MTAB-10640 (https://www.ebi.ac.uk/arrayexpress/experiments/E-MTAB-10640). The expression data is in the database of the National Center for Biotechnology Information Gene Expression Omnibus under the accession GEO: GSE109155, GSE125080 and GSE125079 (https://www.ncbi.nlm.nih.gov/geo/query/acc.cgi?acc=GSE125079 or GSE125080 or GSE109155).
