# Supplementary material for: DNA methylation landscapes from pig’s limbic structures underline regulatory mechanisms relevant for brain plasticity
Source: Sci Rep. 2022 Sep 29;12:16293. doi: 10.1038/s41598-022-20682-x (PMC9522933; doi:10.1038/s41598-022-20682-x)
Supplement: Supplementary file 2 — Supplementary Figures. [file 41598_2022_20682_MOESM2_ESM.pdf]

Supplementary Figures:

DNA methylation landscapes from pig's limbic structures underline regulatory mechanisms relevant for brain plasticity

Alvaro Perdomo-Sabogal[1], Nares Trakooljul[1], Frieder Hadlich[1], Eduard Murani[1], and Klaus Wimmers[1,2]

[1]*Research Institute for Farm Animal Biology (FBN), Institute for Genome Biology, Wilhelm-Stahl-Allee 2, D-18196 Dummerstorf, Germany.*
[2]*Faculty of Agricultural and Environmental Sciences, University Rostock, 18059 Rostock, Germany.*

List of Figures

1 a. Differences in the distribution of the methylation rates across hypothalamus, hippocampus, amygdala, and adrenal gland. b. Principal component analysis using two factors: tissue and sex. 2
2 Protein-protein association STRING network for genes with significantly different meCpGs (q-value < 0.01). 3
3 Enriched ontology terms for biological processes of genes with at least one meCpGs with significant changes in their methylation rate (q-value < 0.01). 4
4 Length of DMRs in nucleotides and number of meCpGs (left panels) and density of meCpGs across DMRs per each pairwise comparison (right panels). 5
5 Unique DMRs across pairwise tissue comparisons. 6
6 Gene ontology enrichment terms for genes harboring a DMRs in their promoter. 7
7 DMR on the promoter of *LHX2* gene and its mRNA tissue expression. 8
8 DMRs on the promoter of *CDH22* and *CEND1* genes and mRNA tissue expression 9
9 DMRs on the promoter of *SKOR2* gene and tissue expression 10
10 DMRs on the promoter of *INSYN1* and *LOXL1* genes. 11

Differences in global methylation rates across tissue comparisons.The boxplot panels show the differences in the distribution of the methylation rates. The difference in their distances was quantified using a two-sample Kolmogorov–Smirnov test. D, the maximum vertical distance between the cumulative distributions. All pairwise differences were significantly different (p-value < 2.2e-16). The scattered plot shows the principal component analysis using mean methylation rates from DMRs, suggesting tissue as the major influencing factor.

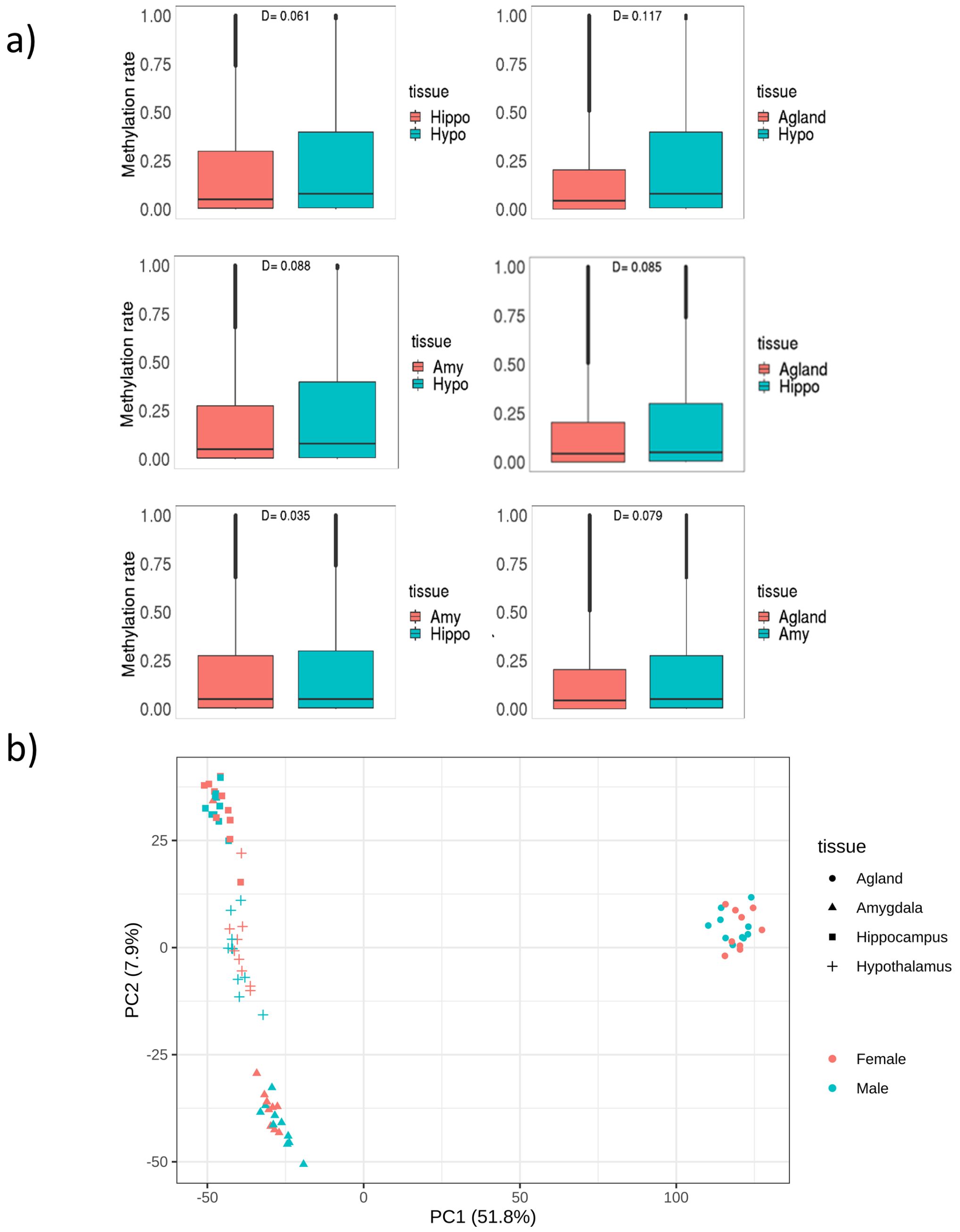

Supplementary Figure 1: a. Differences in the distribution of the methylation rates across hypothalamus, hippocampus, amygdala, and adrenal gland. b. Principal component analysis using two factors: tissue and sex.

This protein-protein association STRING network[1] shows the inter-connectivity between 226 genes that we identified harbor at least one meCpGs their promoter region (TSS ± 2kb) and that exhibit significant changes in the methylation (q-value < 0.01) rate among the hypothalamus, hippocampus, amygdala and adrenal gland from 78 German Landrace piglets. This network reveals a set of genes whose molecular function was enriched for ontology terms associated with direct and indirect DNA gene regulatory functions. To obtain these gene set, we filtered and annotated meCpGs only based on the significance level, independently of the differences in their methylation rate. This network was build using STRING, it shows the full network, but disconnected nodes were hidden. Nodes represent genes, and their color reveals gene association to its regulatory functions. The color of the edges denotes the type of evidence that supports the interaction between pairs of proteins. This network reveals a set of highly interconnected molecular gene regulatory proteins.

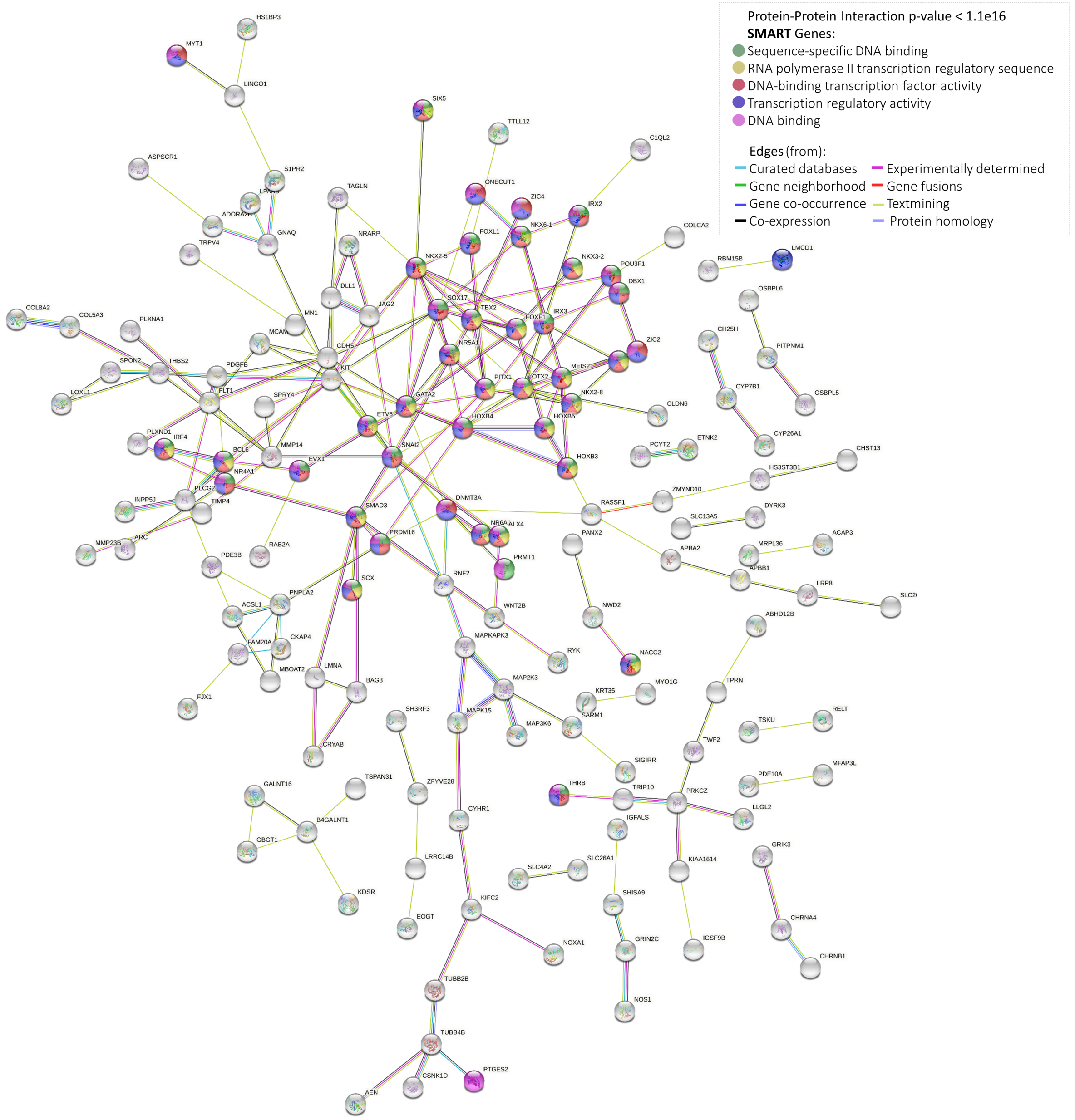

Supplementary Figure 2: Protein-protein association STRING network for genes with significantly different meCpGs (q-value < 0.01).

The structure, nodes interconnectivity, and set up of this STRING network[1] are identical to the supplementary figure S4. However, this network uses a different color code to reveal a set of genes with biological function enriched for terms in the context of organism-, neuro-, and cell development and communication, among others. This network only shows parent GO terms for child terms significantly enriched in this gene set (q-value < 0.01). The whole set of enriched child terms is in the Supplementary Table S7. This network also reveals that a community of genes, mostly TF and other co-regulatory proteins present high protein-protein association connectivity and multiple biological roles (upper part).

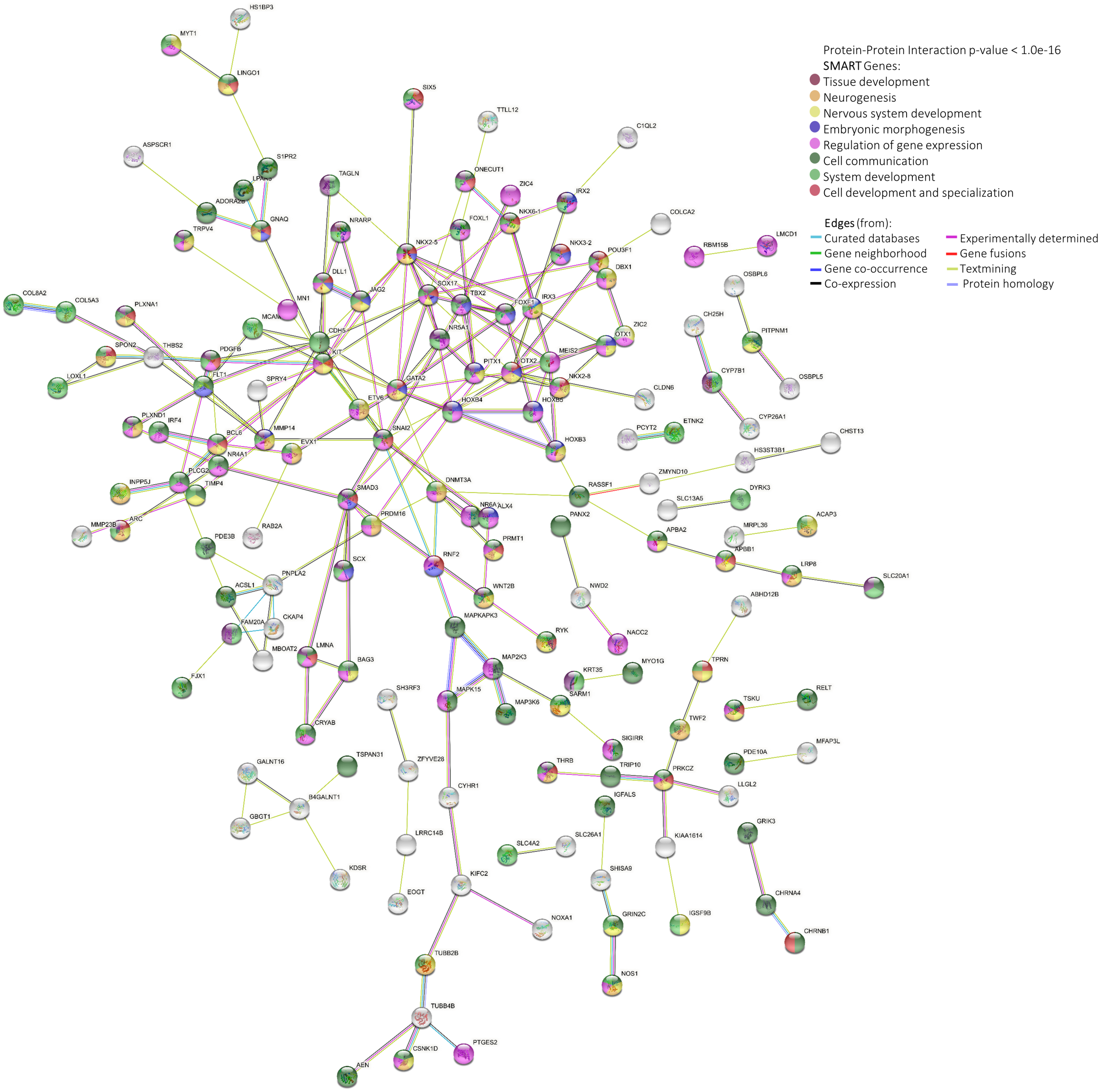

Supplementary Figure 3: Enriched ontology terms for biological processes of genes with at least one meCpGs with significant changes in their methylation rate (q-value < 0.01).

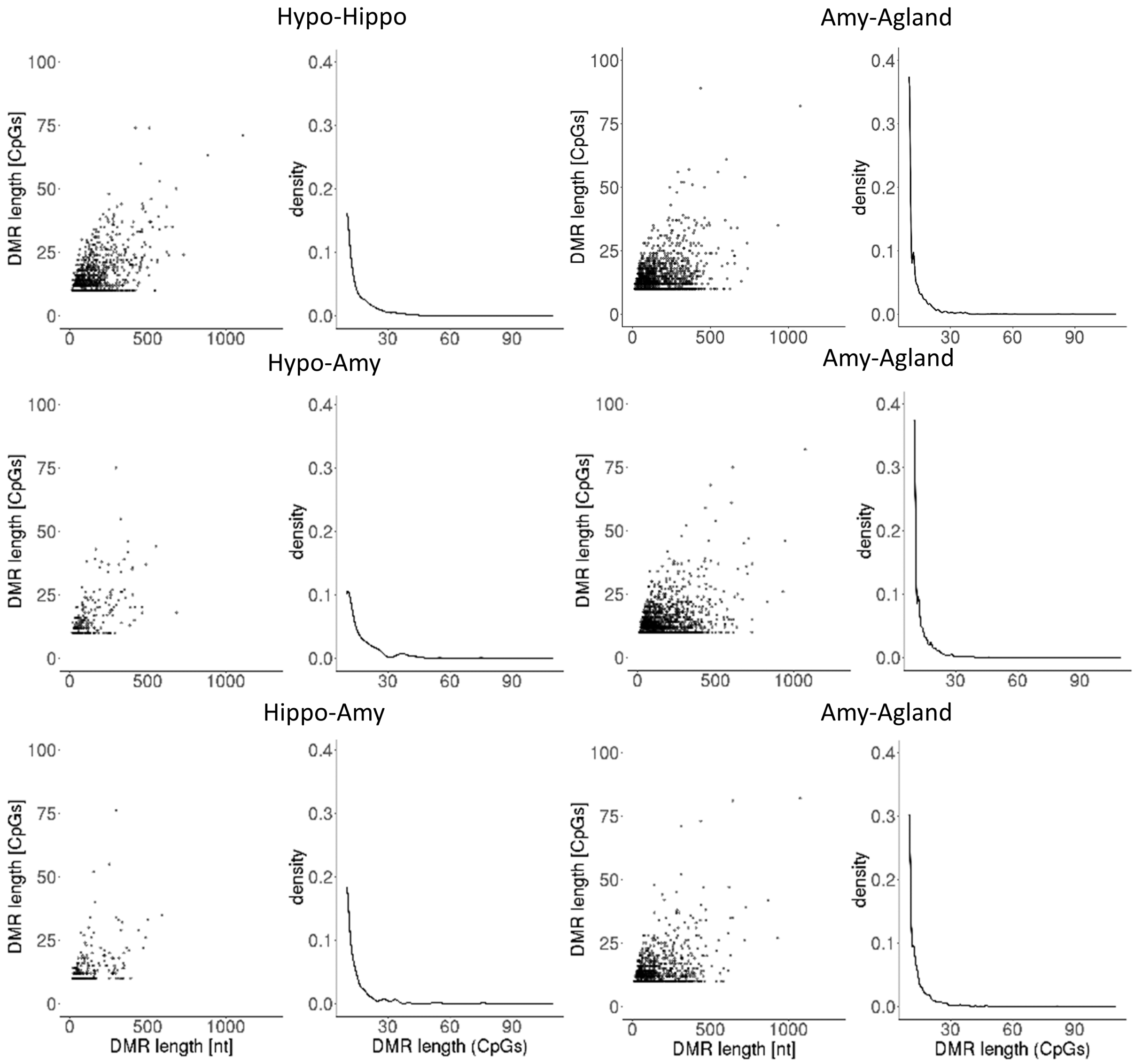

Supplementary Figure 4: Length of DMRs in nucleotides and number of meCpGs (left panels) and density of meCpGs across DMRs per each pairwise comparison (right panels).

Differentially methylated regions that are unique to pairwise comparisons between the hypothalamus (Hypo), hippocampus (Hippo), amygdala (Amy), and adrenal gland (Agland) tissues from German Landrace pigs. Green and blue indicate an increase or decrease in methylation compared across tissues, respectively (>= 0.1, light, >=0.25, dark). This panel shows differences across brain tissues (top row) and among brain tissues and adrenal gland (bottom row).

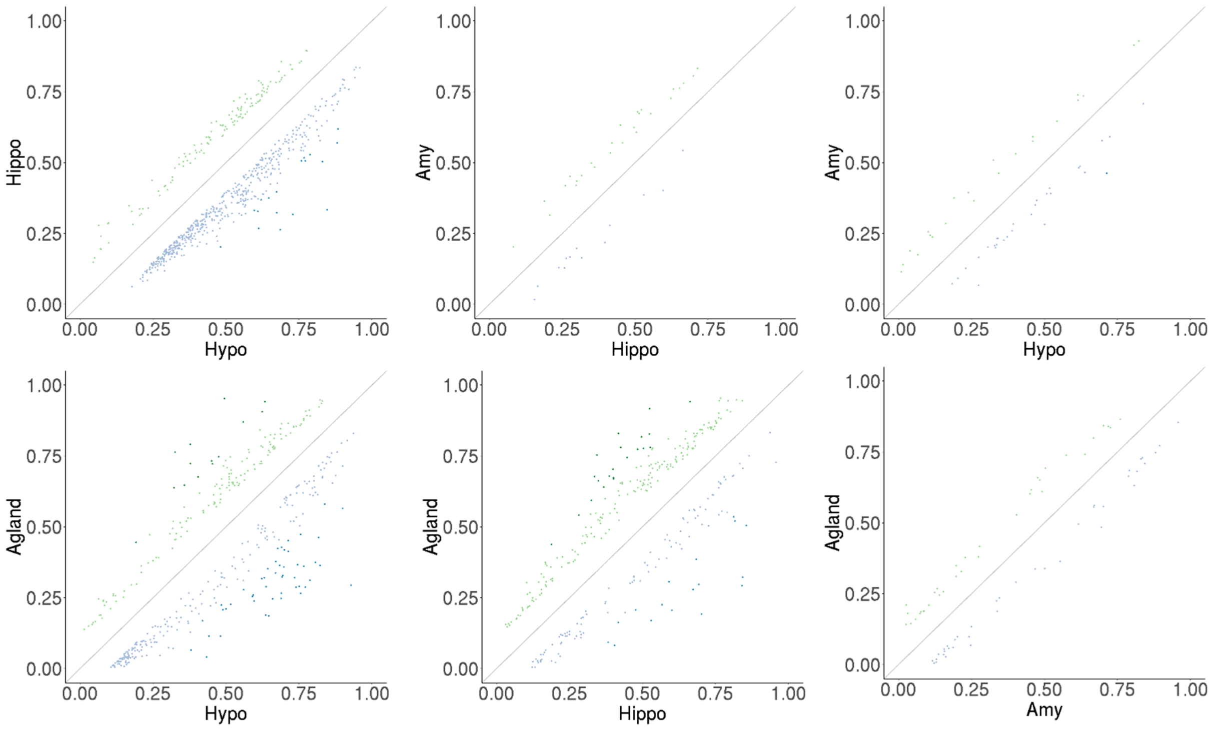

Supplementary Figure 5: Unique DMRs across pairwise tissue comparisons.

Functional annotation of genes harboring at least one DMR in their promoter across pairwise comparisons (only shows enriched terms with a q-value < 0.01). Molecular Function (a) and Biological process terms (b). The size of the bubble represents the significance level, the greater the bubble the smaller the q-value. The color code represents parent terms, and child terms are in the y axis. Missing pairwise comparisons in a and b indicate that enrichment was not detected.

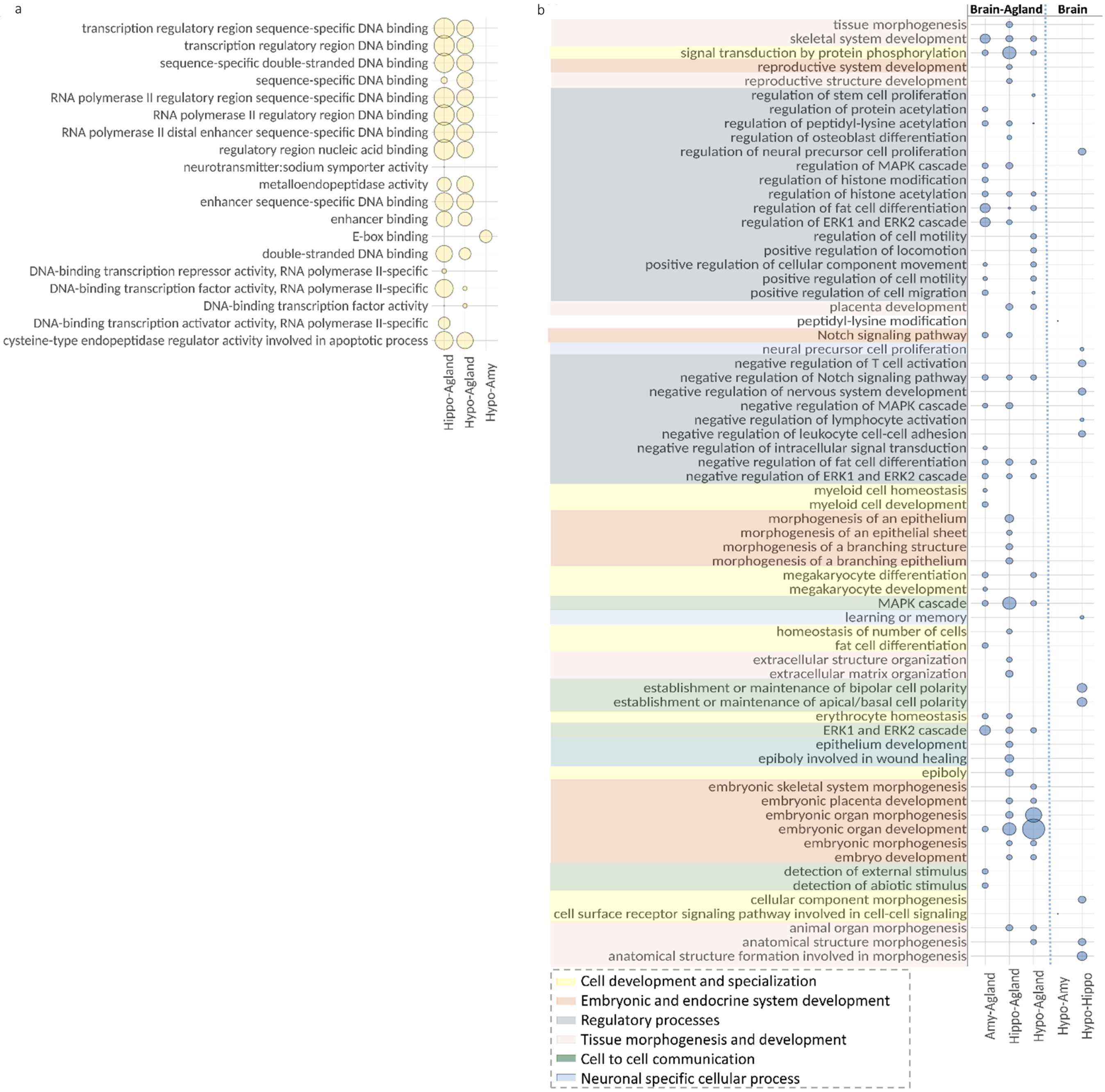

Supplementary Figure 6: Gene ontology enrichment terms for genes harboring a DMRs in their promoter.

DMRs on the promoter region of LIM Homeobox 2 *(LHX2)*. a. Mean methylation rates profiles for hypothalamus, hippocampus, amygdala, and adrenal gland in pig (top). Annotation to genome features (bottom): Differentially methylated regions track (DMRs, red), CpG Islands track (CGI, dark blue), gene annotation to Sscrofa11.1 (light blue), CpG content 100 bp windows (bottom, black).Image built using Integrative genomics viewer (IGV)[2]. b. *LHX2* mRNA expression profile in transcripts per million (TPM) across 54 human tissues. c. *LHX2* Normalized mRNA expression profile (NX) across 14 brain tissues from pig.

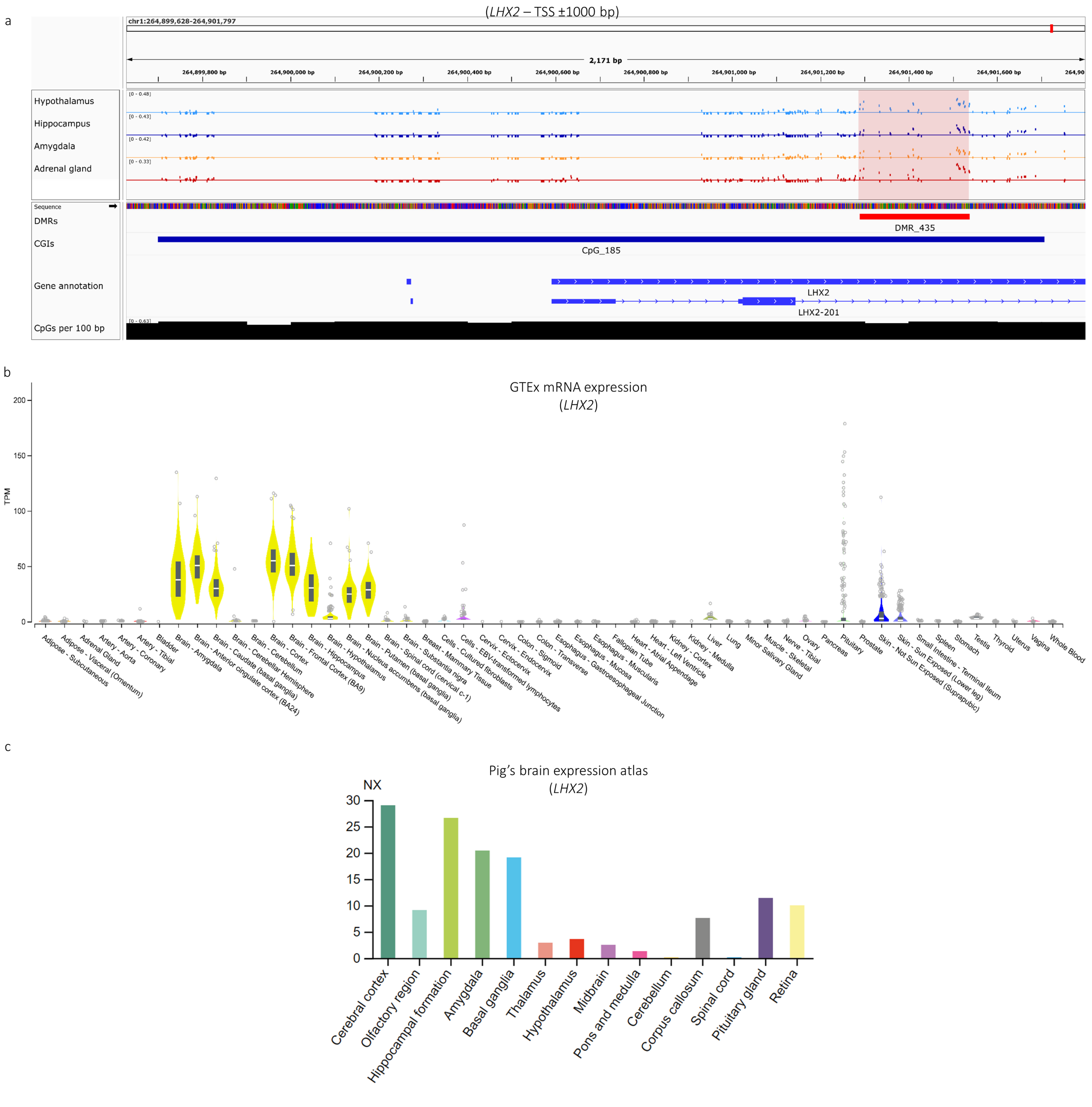

Supplementary Figure 7: DMR on the promoter of *LHX2* gene and its mRNA tissue expression.

DMRs on the promoter region of Cadherin 22 *(CDH22)* and cell cycle exit and neuronal differentiation *CEND1* protein-coding genes. Mean methylation rates profiles for hypothalamus, hippocampus, amygdala and adrenal gland for *CDH22* (a, top) and *CEND1* (b, top) in pig. Annotation to genome features (a and b, bottom), differentially methylated regions track (DMRs, red), CpG Islands track (CGI, dark blue), gene annotation to Sscrofa11.1 (light blue), CpG content 100 bp windows (bottom, black). Image built using IGV[2]. c. *CEND1* mRNA expression profile in transcripts per million (TPM) across 54 human tissues. d. *CEND1* and *CDH22* normalized mRNA expression profile (NX) across 14 brain tissues from pigs.

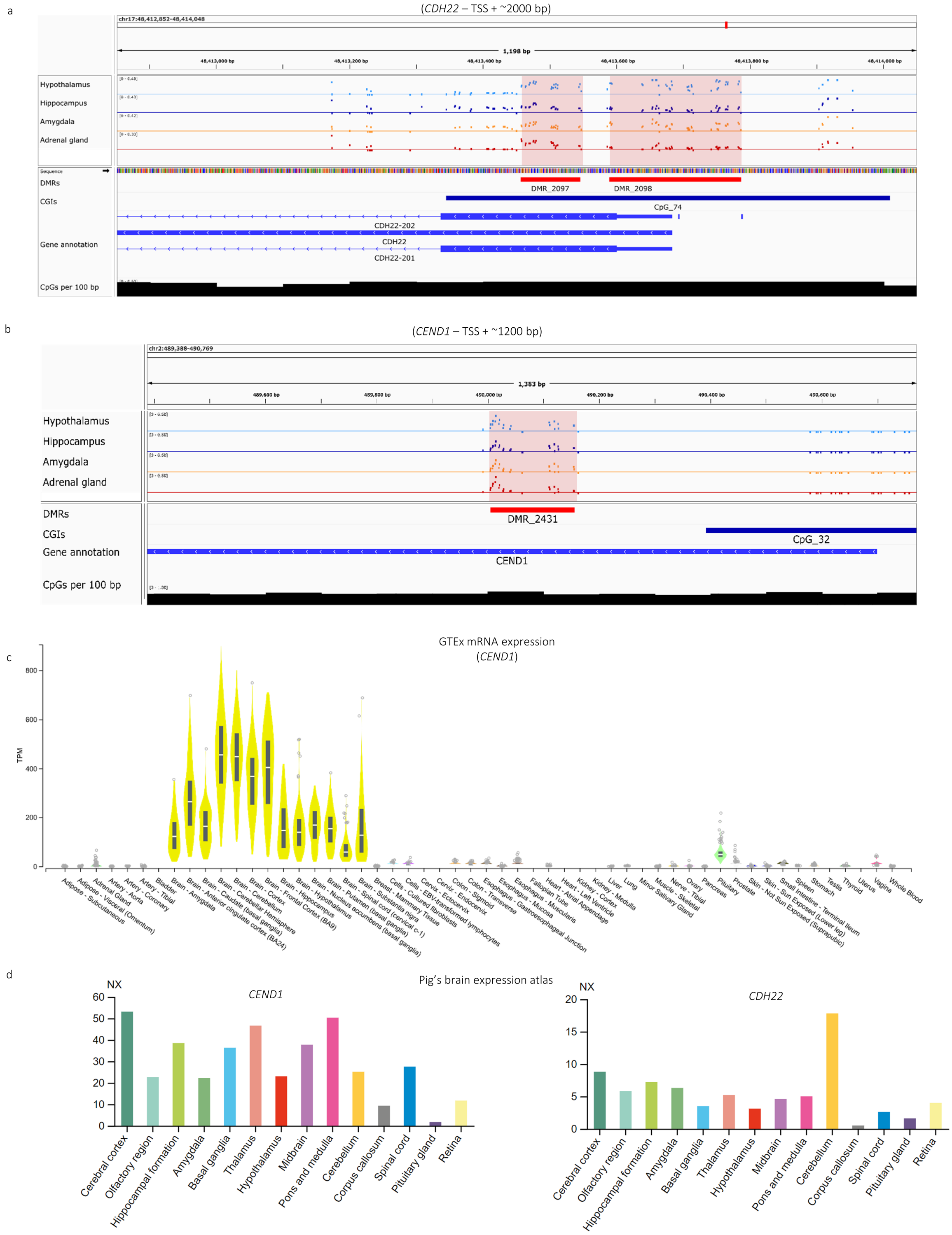

Supplementary Figure 8: DMRs on the promoter of *CDH22* and *CEND1* genes and mRNA tissue expression

DMRs on the promoter region of SKI Family Transcriptional Corepressor 2 *(SKOR2)* protein-coding gene and mRNA tissue expression. a. Mean methylation rates profiles for hypothalamus, hippocampus, amygdala, and adrenal gland in pig (top). Annotation to genome features (bottom): Differentially methylated regions track (DMRs, red), CpG Islands track (CGI, dark blue), gene annotation to Sscrofa11.1 (light blue), CpG content 100 bp windows. Image built using IGV[2]. b. *SKOR2* mRNA expression profile in transcripts per million (TPM) across 54 human tissues. c. *SKOR2* normalized mRNA expression profile (NX) across 14 brain tissues from pig.

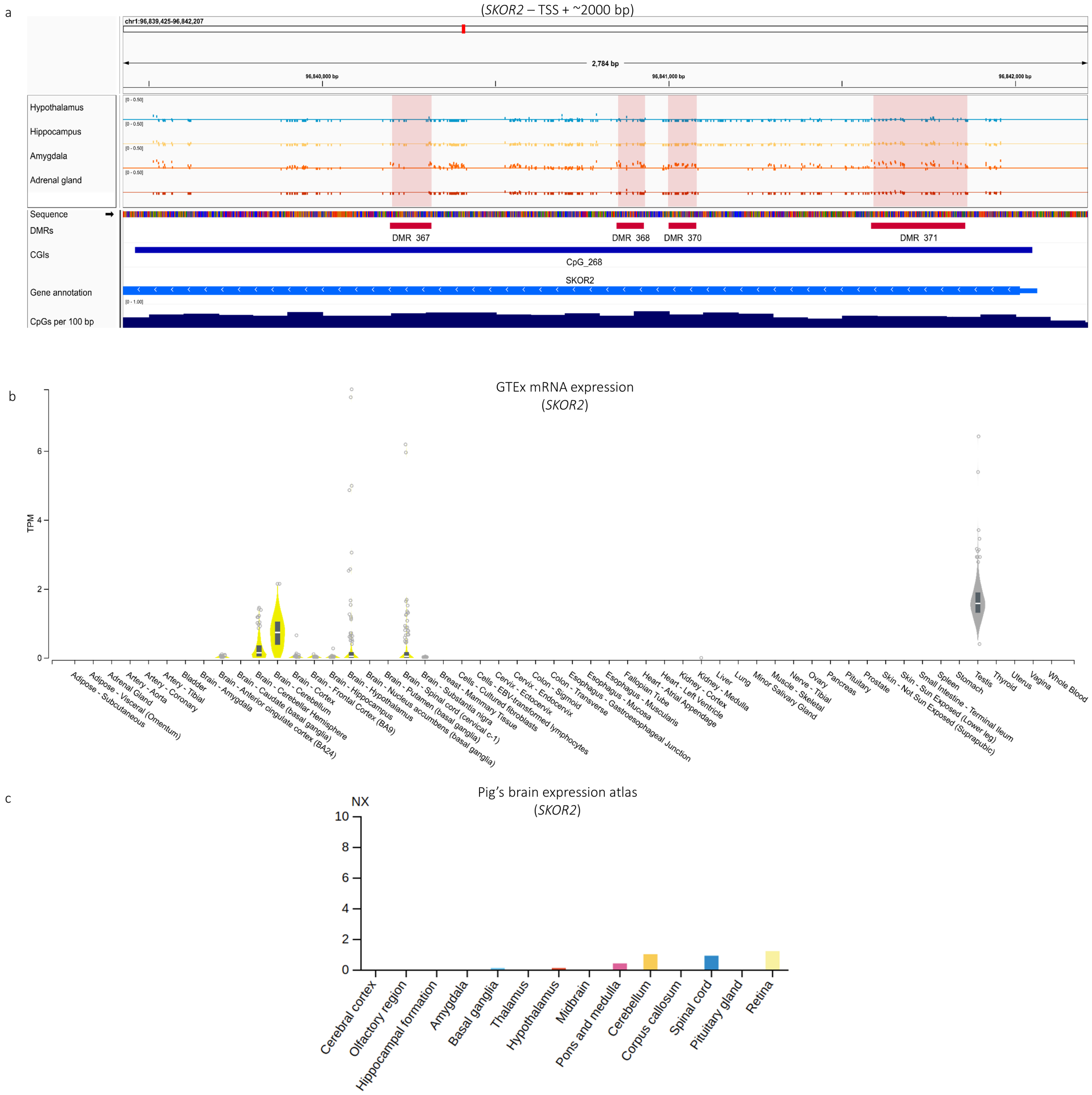

Supplementary Figure 9: DMRs on the promoter of *SKOR2* gene and tissue expression

DMRs on the promoter regions of Inhibitory Synaptic Factor 1 *INSYN1* and the copper ion binding and oxidoreductase activity *LOXL1* protein-coding genes. a. Mean methylation rates profiles for hypothalamus, hippocampus, amygdala, and adrenal gland in pig (top). Annotation to genome features (bottom): Differentially methylated regions track (DMRs, red), CpG Islands track (CGI, dark blue), gene annotation to Sscrofa11.1 (light blue), CpG content 100 bp windows(bottom, black). Image built using IGV[2]. b and c. *INSYN1* and *LOXL1* mRNA expression profiles in transcripts per million (TPM) across 54 human tissues, respectively. d and e. *INSYN1* and *LOXL1* normalized mRNA expression profile (NX) across 14 brain tissues from pigs.

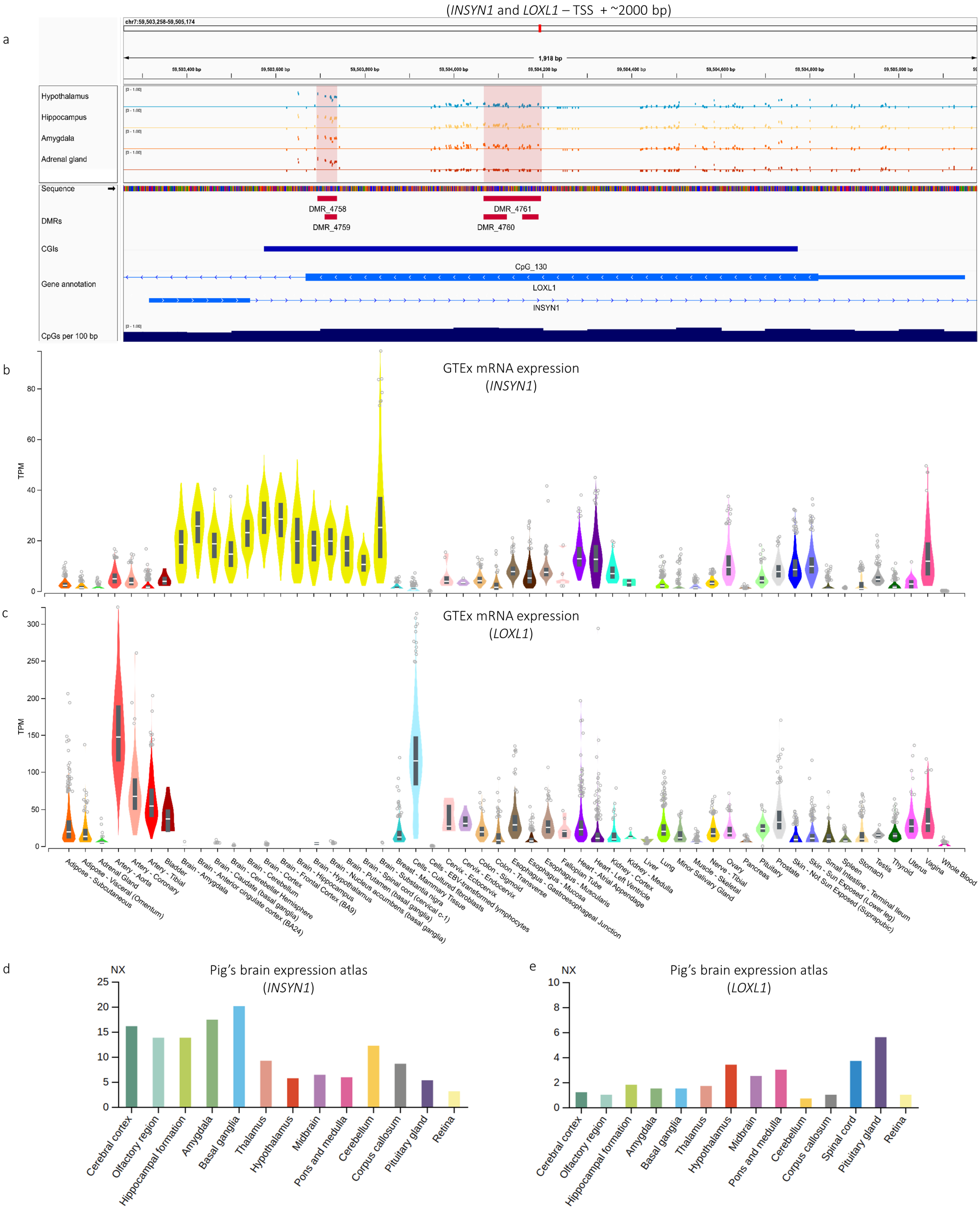

Supplementary Figure 10: DMRs on the promoter of *INSYN1* and *LOXL1* genes.

References

1. D. Szklarczyk, A. L. et al. The string database in 2021: customizable proteinprotein networks, and functional characterization of user-uploaded gene/measurement sets. Nucleic acids research, vol. 49, no. D1, pp. D605–D612(2021).
2. Robinson, J. et al. Integrative genomics viewer. Nat Biotechnol 29, 24–26(2011).
